# Mitofusin2, a rising star in acute‐on‐chronic liver failure, triggers macroautophagy via the mTOR signalling pathway

**DOI:** 10.1111/jcmm.14658

**Published:** 2019-09-26

**Authors:** Ran Xue, Xuemin Zhu, Lin Jia, Jing Wu, Jing Yang, Yueke Zhu, Qinghua Meng

**Affiliations:** ^1^ Department of Critical Care Medicine of Liver Disease Beijing You‐An Hospital Capital Medical University Beijing China

**Keywords:** acute‐on‐chronic liver failure, autophagy, mitofusin2, mTOR

## Abstract

Acute‐on‐chronic liver failure (ACLF) is a life‐threatening syndrome with poor prognosis. Several studies have begun to prove that mitochondria play a crucial role in liver failure. Mitofusin2 (Mfn2) plays a key role in maintaining the integrity of mitochondrial morphology and function. However, the role and underlying mechanisms of Mfn2 on cell autophagy of ACLF remain unclear. Our aim was to explore the effect of Mfn2 on several biological functions involving cell autophagy in ACLF. In this study, we constructed an ACLF animal model and a hepatocyte autophagy model, using adenovirus and lentivirus to deliver Mfn2 to liver cells, in order to assess the effect of Mfn2 on autophagy and apoptosis in ACLF. Furthermore, we explored the biological mechanism of Mfn2‐induced autophagy of ACLF using Western blotting, RT‐PCR and electron microscopy. We found that Mfn2 significantly attenuated ACLF, characterized by ameliorated gross appearance and microscopic histopathology of liver, and reduced serum AST, ALT, and TBIL levels. Mfn2 improved the expressions of LC3‐II, Atg5 and Bcl‐2 and down‐regulated the expression of P62 and Bax in ACLF. Like rapamycin, Mfn2 also significantly inhibited the expressions of p‐PI3K, p‐Akt and p‐mTOR in ACLF. In conclusion, our findings suggest that Mfn2 influences multiple biological functions of ACLF via the PI3K/Akt/mTOR signalling pathway. This study will provide a reliable theoretical basis for the application of Mfn2 as an effective target for ACLF treatment, reversing or delaying the process of ACLF.

## 1 INTRODUCTION

Acute‐on‐chronic liver failure (ACLF) is a life‐threatening syndrome with various manifestations and aetiologies that has a short‐term mortality of 50%‐90%.^1^ Liver transplantation (LT) is the only beneficial and feasible treatment for patients with ACLF.^2^ Shortages of donor livers undoubtedly cause some patients to lose the opportunity for LT. The rapid disease progression and the increasing gap between potential candidates for LT and donor livers available suggest that it is necessary to develop new therapeutic alternatives to prevent the progression of ACLF.^3^

The liver is the central regulator of energy metabolism. Our previous study showed that patients with ACLF have energy metabolism disorders.^4^ Furthermore, energy metabolism disorders contribute to increased mortality risk in patients with ACLF.^5^ Mitochondria play a significant role in energy metabolism. It is known that mitochondrial dysfunction is part of the pathogenesis of liver failure.^6^

Mitochondria are highly dynamic organelles^7^ that are connected to each other in a network that constantly changes in terms of morphological structure. Changes in mitochondrial morphological structure and functional maintenance are achieved through fusion and division processes. As an important member of the mitochondrial fusion protein family, mitofusin2 (Mfn2) plays a key role in the mitochondrial fusion process, regulating mitochondrial function and morphology.^8^

As for the biological function of Mfn2, studies reported that ER stress up‐regulated Mfn2 and that genetic ablation of Mfn2 increased cell death during ER stress.^9^ In skeletal muscle, Mfn2 regulates optimal biological properties via maintaining mitochondrial quality control and efficient mitochondrial metabolism.^10^ With the knockdown of Mfn2 in Hela cells, reduced ATP production, impaired autophagic degradation, diminished cell glycolysis and mitochondrial oxygen consumption rate, and suppressed cell proliferation were observed.^11^ In pancreatic cancer, Mfn2 plays an important role in cell autophagy through the PI3K/Akt/mTOR signalling pathway^12^; that is, Mfn2 regulates the responses of cells to stress, determining cell fate, opening up a broad space in terms of its therapeutic approach.

ACLF is defined as the acute decompensation of liver function in patients with either previously diagnosed or undiagnosed chronic liver disease.^2^, ^3^ Due to the prominent effect of Mfn2 on mitochondrial morphology and functional maintenance, as well as the critical influence of mitochondria in the pathogenesis of ACLF, it is worthwhile to pay more attention to the role of Mfn2 in ACLF. Nevertheless, to date, it remains unclear what the molecular mechanisms of Mfn2 in the pathogenesis of ACLF are.

In this study, we constructed an ACLF animal model and a hepatocyte autophagy model, using adenovirus and lentivirus to deliver Mfn2 to liver cells, in order to assess the effect of Mfn2 on autophagy in ACLF. Furthermore, we explored the biological mechanisms of Mfn2‐induced autophagy of ACLF. This study will provide a reliable theoretical basis for the application of Mfn2 as an effective target for ACLF treatment, reversing or delaying the process of ACLF.

## 2 MATERIAL AND METHODS

### 2.1 Animal models of ACLF

Healthy male Sprague Dawley (SD) rats (150‐170 g) fed in the medical research centre of Beijing You‐an Hospital, Capital Medical University, were used in our experiments. They were acclimated for 2 weeks before experimentation. The ACLF animal model was established according to a published method.^13^ Lentiviruses encoding the Mfn2 (LV‐Mfn2) and control lentivirus were constructed by JI KAI Gene Technology Co. Ltd. Animals were randomly divided into 5 groups (n = 10). The grouping and treatment regimens were as follows: Group I: normal control, the rats receiving normal saline (1.5 mL/Kg), twice per week, 10 weeks; Group II: ACLF model, intraperitoneally receiving vegetable oil and 40% CCL4 mixture (1.5 mL/kg), twice per week, 10 weeks; Group III: LV‐Mfn2 group, receiving vegetable oil and 40% CCL4 mixture (1.5 mL/kg), twice per week, and LV‐Mfn2 (1X10^9^ IU/mL), 200 µL per one rat, once per 3 weeks, 10 weeks; Group IV: LV‐Con group, receiving vegetable oil and 40% CCL4 mixture (1.5 mL/kg), twice per week, and LV‐Con (1X10^9^ IU/mL), 200 µL per one rat, once per 3 weeks, 10 weeks; Group V: LV‐Mfn2 + rapamycin group, receiving vegetable oil and 40% CCL4 mixture (1.5 mL/kg), twice per week, and LV‐Mfn2 (1X10^9^ IU/mL), 200 µL per one rat, once per 3 weeks, beginning from the sixth week, rapamycin (1 mg/kg), once per day, 10 weeks. Groups II–V, against the background of chronic liver injury, were then challenged intraperitoneally with LPS (100 µg/kg) (Sigma Chemical Co.) combined with D‐gal (0.5 g/kg) to induce acute liver failure.

The investigation was conducted according to the ethical standards and the Declaration of Helsinki. This study has also been approved by Beijing You‐an Hospital, Capital Medical University. All methods and procedures including animals were performed according to the guidelines of the Animals Committee.

### 2.2 Hepatocyte autophagy model

The human normal liver HL‐7702 cell line was a gift from the cell laboratory of the Beijing You‐an Hospital, Capital Medical University. The 7702 cells were selected and incubated with 95% air and 5% CO_2_ at a temperature of 37°C (normal control). The Binder three‐gas incubator was used, with a temperature of 37°C, a CO_2_ concentration of 5% and an O_2_ concentration of 0.3% to provide a hypoxic environment, and serum‐free DMEM was used to induce starvation for 6 hours.

### 2.3 Adenovirus‐Mfn2 treatment of cell lines

Adenovirus encoding the Mfn2 open reading frame (Ad‐Mfn2) and control adenovirus were constructed by JI KAI Gene Technology Co. Ltd. The grouping and treatment regimens were as follows: Group I: normal control; Group II: hepatocyte autophagy model; Group III: Ad‐Mfn2 group, where the 7702 cells were incubated with Ad‐Mfn2 at a multiplicity of infection (MOI) of 200 pfu per cell at 37°C for 24 hours, hypoxia and starvation for 6 hours; Group IV: Ad‐Mfn2 NC group, where the 7702 cells were incubated with Ad‐Mfn2 NC at a multiplicity of infection (MOI) of 200 pfu per cell at 37°C for 24 hours, hypoxia and starvation for 6 hours; and Group V: Ad‐Mfn2 + rapamycin group, where the 7702 cells were incubated with Ad‐Mfn2 at a multiplicity of infection (MOI) of 200 pfu per cell at 37°C for 12 hours, rapamycin for 12 hours, hypoxia and starvation for 6 hours.

### 2.4 Subcellular fractionation

The mitochondrial and cytosolic fractions were obtained using a ProteoExtract subcellular proteome extraction kit (Calbiochem).

### 2.5 Western blotting analysis

Western blotting analysis was performed as described previously.^14^ Samples of 50 μg of total protein were used for Western blotting. The list of primary antibodies is shown in Table S1 online. Protein bands were visualized using the SuperSignal West Pico Chemiluminescent Substrate (Thermo Fisher Scientific). The average intensities of each standard protein band were quantified using ImageJ and these results were normalized using GAPDH. The results were column‐plotted using GraphPad Prism 8.0 software.

### 2.6 Electron microscopy

Glutaraldehyde (3%) in 0.2 M sodium cacodylate was used to fix liver tissue samples. The samples were washed three times with PBS. After dehydration using serial concentrations of ethanol, the cells were embedded in Epon. The images were acquired with a transmission electron microscope (JEM‐1200; Jeol Ltd.) at 80 kV. The autophagic vacuole was identified as double‐membrane‐bound vesicle within residual. The quantitative morphometric analysis was also done independently and blindly by two experienced researchers under electron microscopy.^15^

### 2.7 Liver function test

Serum levels of alanine aminotransferase (ALT), aspartate aminotransferase (AST) and serum total bilirubin (TBIL) were measured with an AU400 automatic biochemical analyser.

### 2.8 Haematoxylin‐Eosin (H&E) Staining

Livers were fixed in 10% buffered formalin, dehydrated with graded ethanol and embedded in paraffin for sectioning. Five‐micron paraffin sections were mounted on glass slides, rehydrated with distilled water and stained with haematoxylin and eosin for light microscopy examination. Routine H&E staining was performed as described previously.^16^ The staining results were assessed independently and blindly via two experienced pathologists under light microscope.

### 2.9 Quantitative real‐time RT‐PCR

Guided by the manufacturer's instructions, an RNeasy Mini Kit (QIAGEN) was used to isolate total RNA. The RT‐PCR assay was performed as described previously.^16^ GAPDH gene was used as an internal standard. The list of primers is shown in Table S2 online.

### 2.10 Statistical analyses

Statistical significance was calculated via Student's *t* test (SPSS 19.0; SPSS Inc). Quantitative variables are shown as the mean ± SD on the basis of at least three separate experiments. *P* < .05 was considered significant.

## 3 RESULTS

### 3.1 Morphological characteristics of the liver

**Figure 1:**
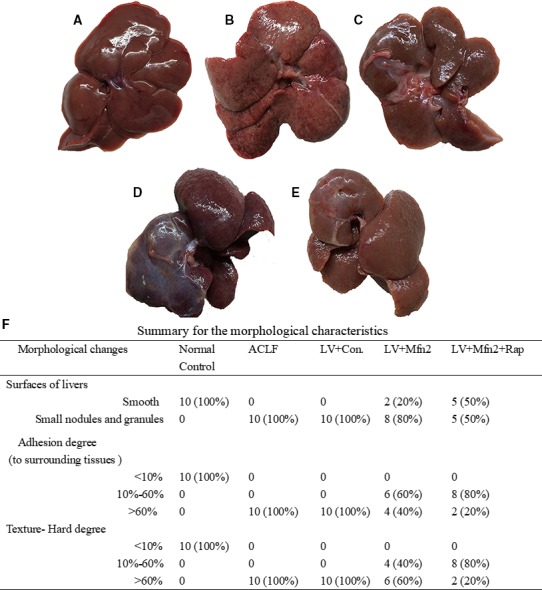
Morphological characteristics of the liver. A, The normal control group; B, ACLF group; C, LV‐Mfn2 group; D, LV‐Mfn2 negative control group; E, LV‐Mfn2 + Rap group; F, Summary for the detailed morphological characteristics of liver

In the normal control group (Group I), the livers were rosy and smooth with intact lobule structure and no adhesions to surrounding tissues (Figure 1A). In the ACLF group (Group II) and the LV‐Mfn2 negative control group (Group IV), liver tissues were congested and hard, with severe adhesions to surrounding tissues and small nodules and granules on the surface (Figure 1B,D). In the LV‐Mfn2 (Group III) and LV‐Mfn2 + Rap groups (Group V), the surfaces of livers were smoother, softer in texture and less adherent to the surrounding tissue (Figure 1C,E). The summary for the detailed morphological characteristics of liver is shown in Figure 1F.

### 3.2 Characteristics of HE staining in the liver

**Figure 2:**
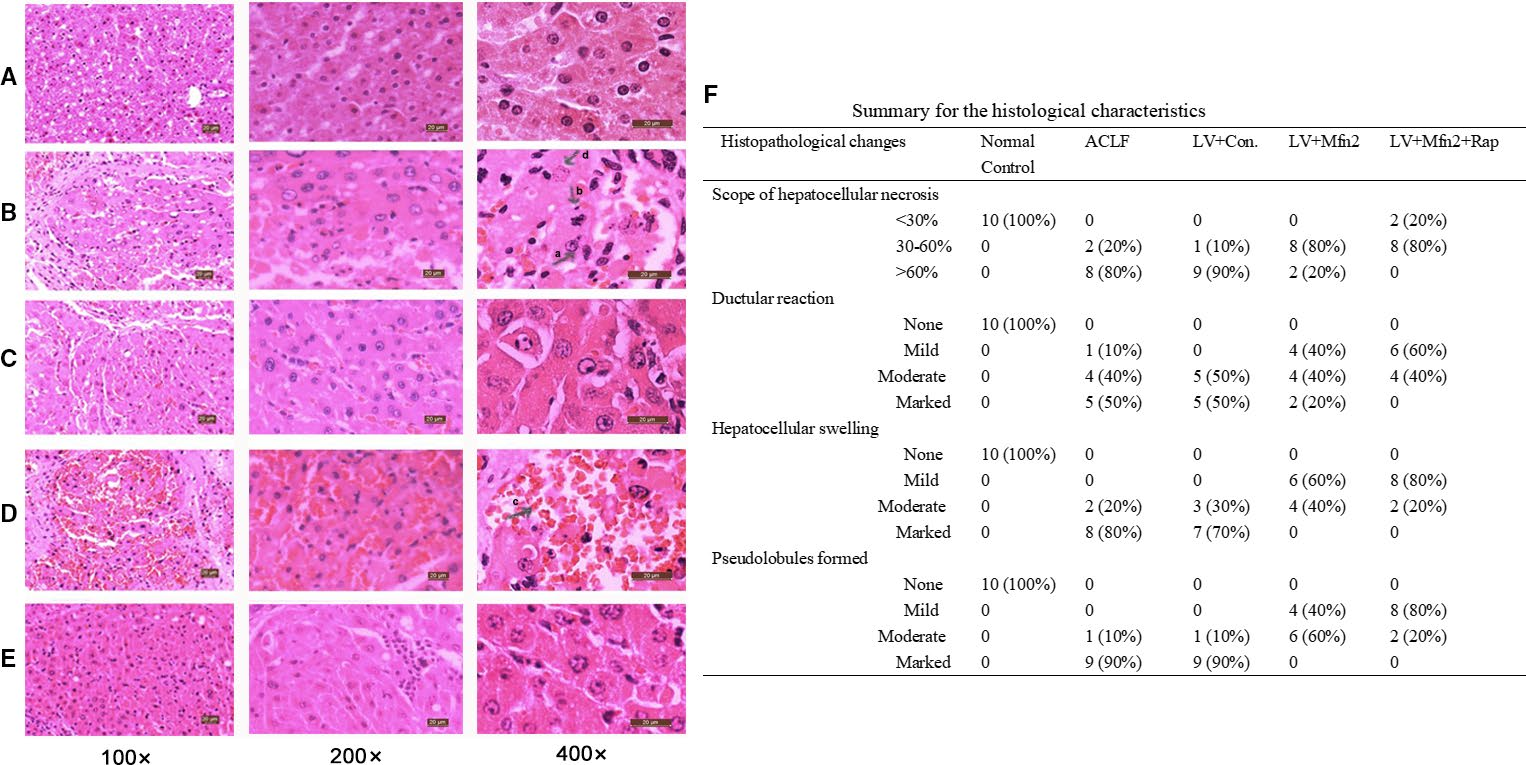
Characteristics of HE staining in the liver. A, The normal control group; B, ACLF group; C, LV‐Mfn2 group; D, LV‐Mfn2 negative control group; E, LV‐Mfn2 + Rap group. a, liver cell; b, inflammatory cell infiltration; c, red cell; and d, Kupffer cell. F, Summary for the histological characteristics in the liver

Compared with the normal control group (Group I), other groups showed various degrees of damage. In the ACLF group (Group II) and the LV‐Mfn2 negative control group (Group IV), the lobular structure collapsed, with a large number of fibrous tissues proliferated and pseudolobules formed. The hepatocytes showed massive fused necrosis, with the necrotic cells disappearing and the surrounding inflammatory cells infiltrated substantially (Figure 2B,D). In the LV‐Mfn2 group (Group III), the hepatic lobule structure was basically intact, with occasional pseudolobules formed, showing small amounts of necrosis, cell balloon‐like changes or vacuolar degeneration (Figure 2C). The LV‐Mfn2 + Rap group (Group V) had a milder degree of fibrosis, intact hepatic lobule structure and occasional small focal necrosis; hepatic cord and hepatic sinus structure were basically intact (Figure 2E). The pathological results showed that Mfn2 overexpression had a protective effect on ACLF. With Mfn2 overexpression, the addition of rapamycin significantly reduced the degree of ACLF injury and improved the liver status of ACLF. The summary for the detailed histological characteristics in the liver is shown in Figure 2F.

### 3.3 Effect of Mfn2 on liver function in ACLF

As shown in Table 1, compared with the normal control group (Group I), the levels of ALT, AST and TBIL»in the other groups increased, indicating that the liver function of each treatment group (Groups II–V) was damaged. The ALT, AST and TBIL in the LV‐Mfn2 group (Group III) were lower than that of the ACLF group (Group II) and the LV‐Mfn2 negative control group (Group IV) (*P* < .05), suggesting that Mfn2 overexpression attenuated the degree of liver injury in ACLF. The liver function indexes of the LV‐Mfn2 + Rap group (Group V) were significantly better than those of the other treatment groups (*P* < .05), suggesting that rapamycin‐activated autophagy during Mfn2 overexpression significantly improved liver function in ACLF.

**Table 1:** Effect of Mfn2 on liver function in ACLF

Groups	ALT (U/L)	AST (U/L)	TBIL (mg/dL)	Ln(ALT)	Ln(AST)
Normal control group	59.5 ± 9.43	201.73 ± 22.67	0.63 ± 0.21	4.08 ± 0.15	5.30 ± 0.16
ACLF group	9902.73 ± 5590.36	14 413.27 ± 10 599.05	29.17 ± 4.92*	9.10 ± 0.54*	9.41 ± 0.69*
LV‐Mfn2 group	3744.27 ± 2414.03	4746.43 ± 2134.27	18.27 ± 2.35*	8.05 ± 0.79*	8.36 ± 0.62*
LV‐Con group	15 901.47 ± 2721.83	17 415.97 ± 3298.48	29.80 ± 6.49*	9.67 ± 0.17*	9.75 ± 0.18*
LV‐Mfn2* + *Rap group	137.47 ± 230.72	2667.90 ± 1432.62	8.63 ± 2.65*	6.93 ± 0.24*	7.40 ± 0.26*

* *P* < .05.

### 3.4 Effect of Mfn2 on apoptosis in ACLF

**Figure 3:**
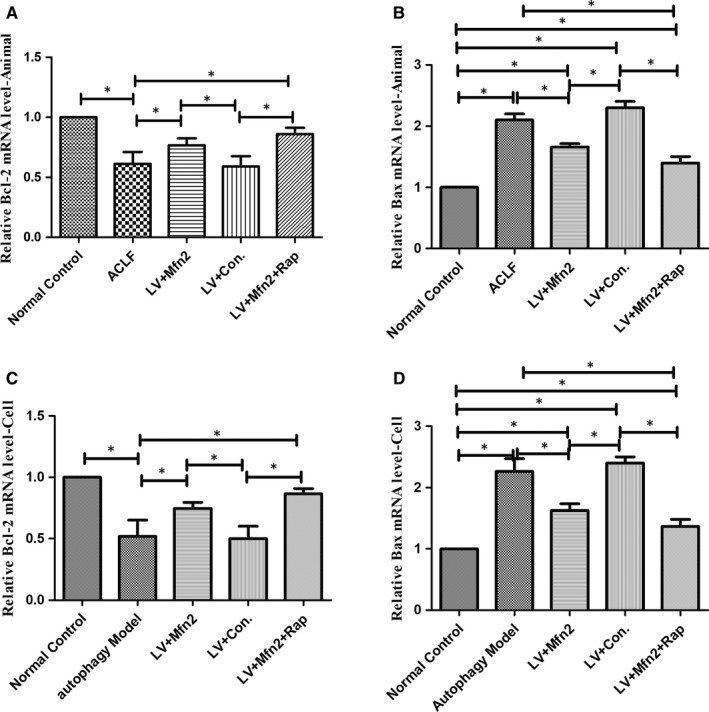
Effect of Mfn2 on apoptosis in ACLF. A, In the ACLF animal model, the expression of Bcl‐2 mRNA levels; B, in the ACLF animal model, the expression of Bax mRNA levels; C, in the hepatocyte autophagy model, the expression of Bcl‐2 mRNA levels; and D, in the hepatocyte autophagy model, the expression of Bax mRNA levels

In the ACLF animal and hepatocyte autophagy models, for assessing apoptosis with Mfn2 overexpression, the expression of Bcl‐2 and Bax was measured using RT‐PCR analysis. Bax levels were significantly lower in the Mfn2 overexpression group. In addition, Mfn2 significantly increased Bcl‐2 levels compared with the ACLF group. Compared with the Mfn2 overexpression group, combined Mfn2 and rapamycin administration further decreased apoptosis (Figure 3).

### 3.5 The effect of Mfn2 on cell autophagy in ACLF

**Figure 4:**
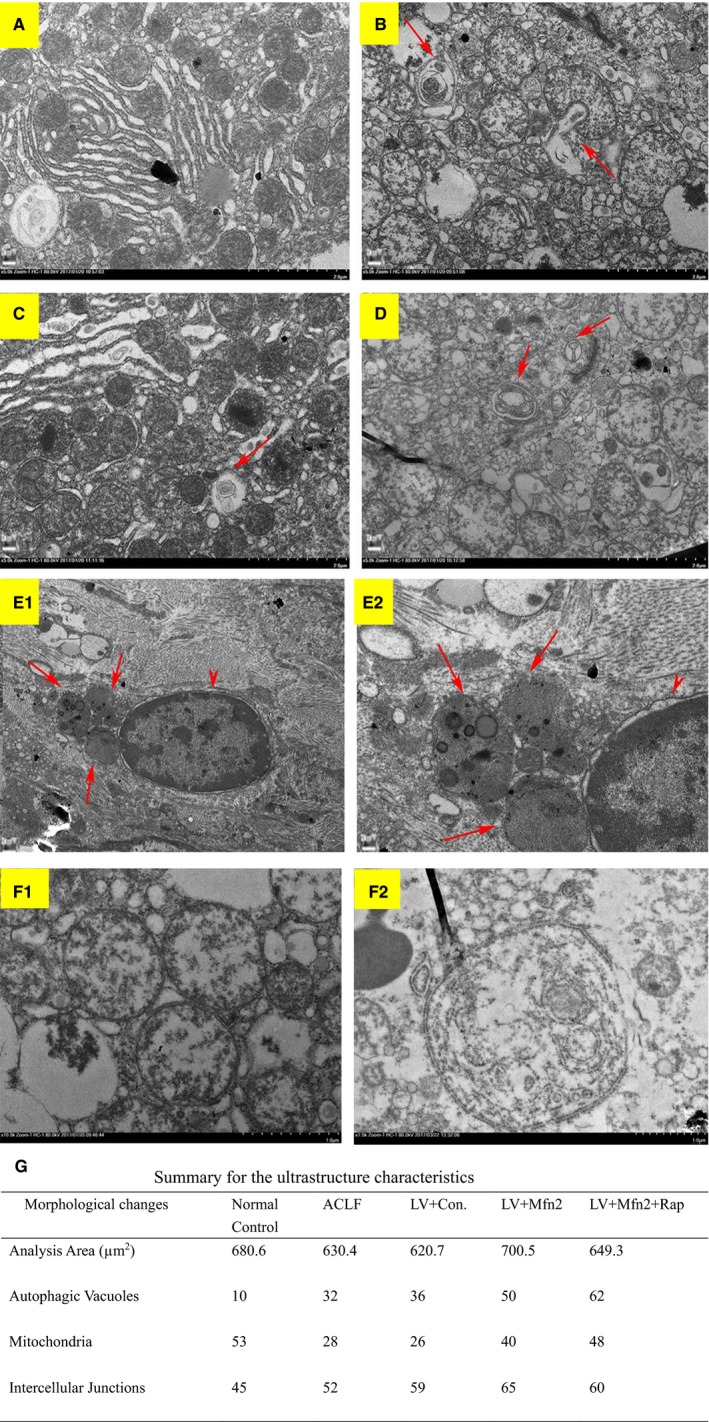
Ultrastructure of the liver via electron microscopy. A, The normal control group (original magnification, x5000); B, ACLF group (original magnification, x5000); C, LV‐Mfn2 group (original magnification, x5000); D, LV‐Mfn2 negative control group (original magnification, x500x); E‐1, LV‐Mfn2 + Rap group (original magnification, x2500); E‐2, LV‐Mfn2 + Rap group (original magnification, x6000) (long arrows: autophagosomes; short arrows: nuclear apoptotic signs); F‐1, mitochondrial morphological changes in the ACLF group (original magnification, x10000); F‐2, mitochondrial morphological changes in the LV‐Mfn2 group (original magnification, x5000); G, quantitative analysis of electron microscopy images. The number of mitochondria, autophagic vacuoles and intercellular junctions was counted from six images. The numbers represent the sum of counts obtained from six images

Electron microscopy was used as the gold standard for ultrastructure. In the ACLF animal model, under the electron microscope, the normal control group (Group I) had normal liver cell morphology, with rounded nuclei, scattered nuclear inclusions, clear nuclear membranes, as well as abundant mitochondria, endoplasmic reticulum and other organelles in the cytoplasm, without autophagosomes (Figure 4A). The ACLF model group (Group II) and the LV‐Mfn2 negative control group (Group IV) showed severe apoptosis with irregular shape and pyknosis of the nuclei. A large number of autophagosomes or autophagic lysosomes were observed (Figure 4C). Compared with Groups II and IV, the LV‐Mfn2 group (Group III) had relatively intact nuclei, mitochondria and endoplasmic reticulum, with less apoptosis and more autophagosomes, suggesting that Mfn2 overexpression promoted autophagy so as to protect hepatocytes. The LV‐Mfn2 + Rap group (Group V) had more complete hepatocyte structure than did the LV‐Mfn2 group (Group III), with a large amount of autophagosomes or autophagic lysosomes (Figure 4E). Furthermore, in the LV‐Mfn2 + Rap group (Group V), the typical phenomenon of apoptosis can be seen when autophagy is excessive (Figure 4E). Quantitative analysis of electron microscopy images captured is shown in Figure 4G.

We also observed mitochondrial morphological changes under electron microscopy (Figure 4F). Compared with the ACLF group, autophagosomes mainly appeared in the cytoplasm, and there was no significant difference for the count of mitochondria enveloped by autophagic vesicles after LV‐Mfn2 treatment in ACLF.

**Figure 5:**
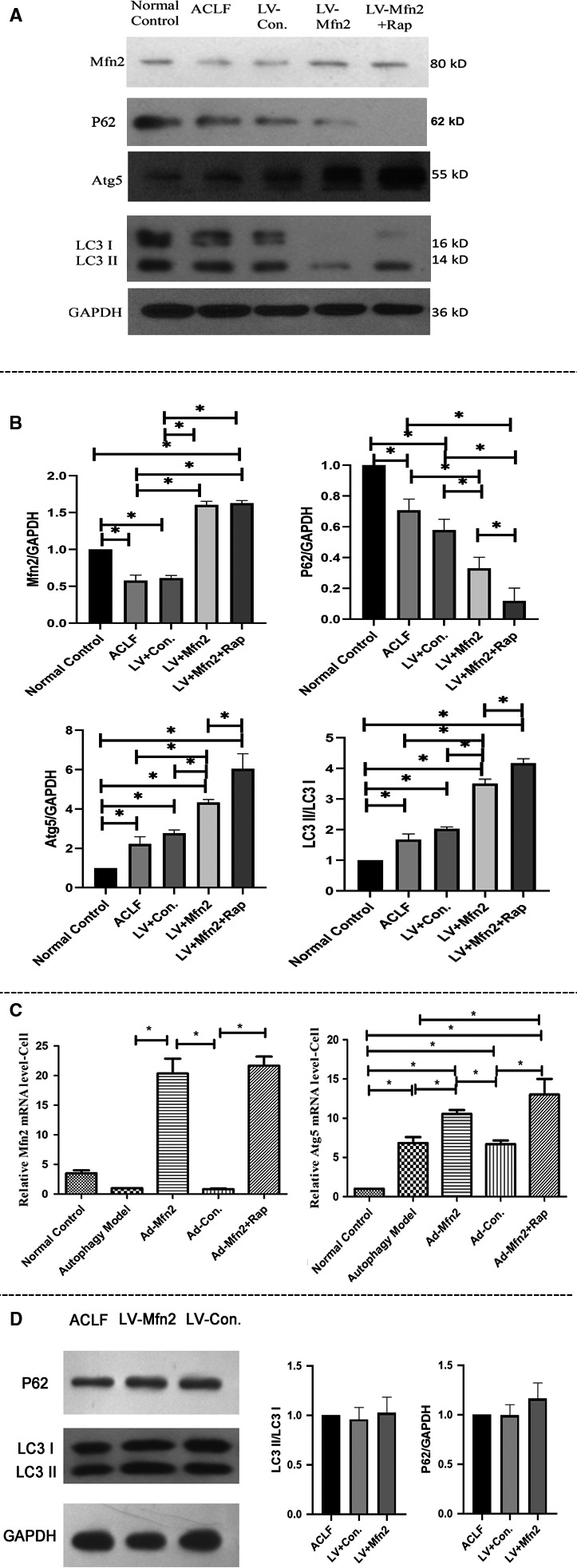
Effect of Mfn2 on autophagy in ACLF. A, Western blotting analysis for the expressions of P62, Mfn2, LC3‐II/I and Atg5 in vivo; B, quantification of Western blots for P62, Mfn2, LC3‐II/I and Atg5 expressions in the different groups (*P < .05. N = 3); C, RT‐PCR analysis for the expressions of Mfn2 and Atg5 in the hepatocyte autophagy model (*P < .05. N = 3); D, Western blotting analysis for the expressions of P62 and LC3‐II/I in mitochondrial fractions in vivo (N = 3)

In the ACLF animal model, Western blotting analyses showed that the expressions of LC3‐II/LC3‐I and Atg5 were greater in the LV‐Mfn2 group (Group III) than in the ACLF group (Group II). In the LV‐Mfn2 + Rap group (Group V), there were further significantly greater expressions of LC3‐II/LC3‐I and Atg5. Meanwhile, there were also less expressions of Mfn2 and P62 in the ACLF group (Group II) than in the normal control group (Group I) (Figure 5A). In the hepatocyte autophagy model, there were the same effects of Mfn2 on autophagy via RT‐PCR analysis (Figure 5C).

Meanwhile, the Western blots were also performed with mitochondrial fractions in the ACLF animal model. Compared with the ACLF group, there was no significant difference for the expressions of LC3 II/I and P62 in the LV‐Mfn2 group (Figure 5D). All these data indicated that Mfn2 mainly promotes autophagy instead of mitophagy during ACLF.

### 3.6 Mfn2 enhances cell autophagy via the PI3K/AKT/mTOR signalling pathway

**Figure 6:**
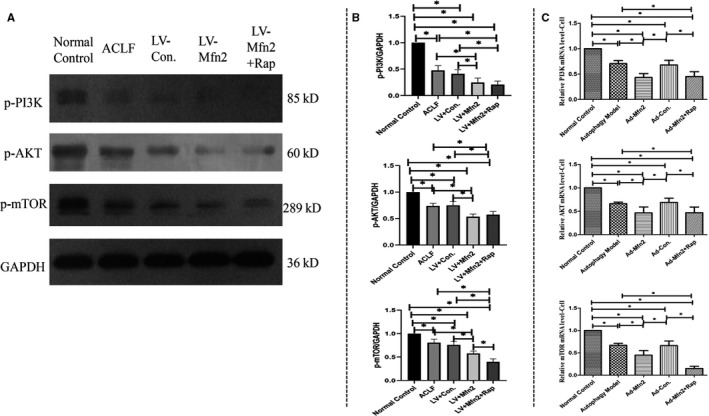
A, Western blotting analysis for the expressions of p‐PI3K, p‐AKT and p‐mTOR in vivo. B, Quantification of Western blots for p‐PI3K, p‐AKT and p‐mTOR expressions in the different groups (*P < .05. N = 3). C, RT‐PCR analysis of the expressions of PI3K, AKT and mTOR in the hepatocyte autophagy model (*P < .05; N = 3)

Next, in the ACLF animal model, Western blotting analyses showed that Mfn2 significantly decreased the expression of phosphorylated‐PI3K (p‐PI3K), phosphorylated‐Akt (p‐Akt) and phosphorylated‐mTOR (p‐mTOR) (Figure 6A). Given that rapamycin induces autophagy by inhibiting mTOR expression, we believe that like rapamycin, Mfn2 induces autophagy via the PI3K/AKT/mTOR signalling pathway. In the hepatocyte autophagy model, there were similar results of Mfn2 on autophagy via the PI3K/AKT/mTOR signalling pathway, according to RT‐PCR analysis (Figure 6C).

## 4 DISCUSSION

Recently, Mfn2 has become a rising star in the application and prospect of mitotherapy among mitochondria‐related diseases.^17^, ^18^ It is known that mitochondrial dysfunction participates in the pathogenesis of ACLF.^6^ In this study, we first proposed that Mfn2 increased autophagy via the PI3K/AKT/mTOR signalling pathway in ACLF. Mfn2 is also believed to perform anti‐apoptotic functions in ACLF. Meanwhile, Mfn2 significantly reduced the degree of ACLF injury and improved the liver status of ACLF. Finally, the data suggest that Mfn2 can be a potential clinical therapeutic target in ACLF.

Autophagy is a self‐digestive process that takes part in various situations, including the destruction of intracellular pathogens, nutrient starvation and degradation of damaged organelles.^19^ Autophagy is a unique mechanism for the decomposition of protein aggregates and large organelles. Therefore, autophagy can ensure cell survival under stressful conditions and maintain cell homoeostasis.^20^ Owing to its role in protein and carbohydrate storage and high biosynthetic activity, hepatocytes could be particularly dependent on autophagy. In the liver, autophagy suppresses lipid accumulation, protein aggregates, chronic cell death, oxidative stress and inflammation.^21^ In our study, we found that interventions in hepatic autophagy can delay disease progression and reduce liver damage of ACLF. Meanwhile, Mfn2 as a rising star in ACLF triggers autophagy via the PI3K/Akt/mTOR signalling pathway to alleviate liver injury.

Hepatic apoptosis is thought to be a principal pathological feature of ACLF. Apoptosis and autophagy are two interconnected mechanisms subsequent to cellular stress.^21^ To some degree, autophagy acts to create a cellular milieu in which survival is favoured. In fact, autophagy triggers pro‐survival mechanisms. Therefore, autophagy counteracts apoptotic cell death by the cell survival pathway.^22^ In our study, Mfn2 also exerted anti‐apoptotic function in ACLF. Autophagy may be the trigger of anti‐apoptotic function of Mfn2 in ACLF. Furthermore, anti‐apoptotic function may be a mechanism related to the protective role of Mfn2 in ACLF.

Mitochondria are highly dynamic organelles, responding to cellular stress by changes in interconnectedness, overall mass and subcellular localization.^23^ The changes in overall mitochondrial mass reflect the balance between the rates of mitophagy and mitochondrial biogenesis.^24^ Mfn2 plays an important role in the mitochondrial dynamic fission. In our study, Mfn2 was associated with a protective role in ACLF. The data also indicated that mitochondrial dynamics may be a key therapeutic strategy for ACLF.

This study also has some limitations. Further studies concerning knockdown experiments of Mfn2 in ACLF are required in the future. Meanwhile, the clinical expression of Mfn2 is an important demonstration of the protective role of Mfn2 in ACLF.

## 5 CONCLUSION

Our findings suggest that Mfn2 influences several biological functions in ACLF via the PI3K/Akt/mTOR signalling pathway. The biological function of Mfn2 in ACLF was enhanced by the addition of rapamycin. Mfn2 can act as a therapeutic target in ACLF treatment.

## CONFLICT OF INTEREST

The authors declare no conflict of interest.

## DISCLOSURES

No potential conflict (financial, professional or personal) that is relevant to the manuscript.

## AUTHOR CONTRIBUTIONS

RX wrote this manuscript. QHM conceived the idea for this study. YKZ and JW analysed the data. XMZ, JW and JY did the experiments. RX and XMZ contributed equally to this work.

## Data Availability

The raw data supporting the conclusions of this manuscript will be made available by the authors, without undue reservation, to any qualified researcher.
