# Supplementary material for: Mitofusin2, a rising star in acute‐on‐chronic liver failure, triggers macroautophagy via the mTOR signalling pathway
Source: J Cell Mol Med. 2019 Sep 26;23(11):7810–8. doi: 10.1111/jcmm.14658 (PMC6815802; doi:10.1111/jcmm.14658)
Supplement: Supplementary file 1 [file JCMM-23-7810-s001.docx]

Supplementary Table S1 online.

Antibodies and conditions used for western blotting analyses.

| Antibody | Number | Species | Dilution | Source |
| --- | --- | --- | --- | --- |
| Atg5 | #12994 | Rabbit monoclonal antibody | 1:1000 | Cell Signaling Technology |
| LC3B | L7543 | Rabbit polyclonal antibody | 1:1000 | Sigma |
| P62 | #39749 | Rabbit monoclonal antibody | 1:1000 | Cell Signaling Technology |
| GAPDH | #5174 | Rabbit polyclonal antibody | 1:1000 | Cell Signaling Technology |
| Phospho-mTOR (Ser2448) | #2971 | Rabbit polyclonal antibody | 1:1000 | Cell Signaling Technology |
| Phospho-Akt (Ser473) | #4060 | Rabbit monoclonal antibody | 1:1000 | Cell Signaling Technology |
| Mitofusin-2 | #9482 | Rabbit monoclonal antibody | 1:1000 | Cell Signaling Technology |
| PI3 Kinase p85 (19H8) | #4257 | Rabbit monoclonal antibody | 1:1000 | Cell Signaling Technology |

Supplementary Table S2 online.

Sequences of the primers used in this study

| Primer* | 5′-3′ | | 5′-3′ | |  |
| --- | --- | --- | --- | --- | --- |
| Mfn2-RT | TTGGCTTTGCTCTGAAGTGA | | CTGGGACATTTGCTCATCTG | |  |
| PI3K-RT | ACCAGCACTGCCTCCTAAAC | | TCTTCATCATCTTCCACCAGTG | |  |
| AKT-RT | ACACAATCTCCGCACCGTA | | ACTCATTCCAGACCCACGAC | |  |
| mTOR-RT | TTGTGCTCTGGATTGAGGTG | | GGAATGCTGGTGTCCTTTGT | |  |
| Atg5-RT | CTCTGCCTTGGAACATCACA | | AGCGTCAGCTTCCTTCACAC | |  |
| GAPDH-RT | GGGTGTGAACCACGAGAAAT | | ACTGTGGTCATGAGCCCTTC | |  |
| Bax-RT | | GAGGCAACCTGACCAGAAAC | | ATGATGGCAGTGGAGGAAAG | |
| Bcl2-RT | | CTTGATTGAGCGAGCCTTTC | | TGGACTGCCCCAGAAAAATA | |

*Eight sets of primers were designed using the Primer Explorer version 4 software (Eiken Chemical Co., Ltd., Tokyo, Japan; http://primerexplorer.jp/elamp4.0.0/index.html) and synthesized by Shanghai Sangon Co., Ltd.
